# Arthroscopic Physeal Sparing Anterior Cruciate Ligament Reconstruction and Lateral Extra-articular Tenodesis With Semitendinosus and Gracilis Tendons

**DOI:** 10.1016/j.eats.2023.09.018

**Published:** 2024-01-01

**Authors:** Edoardo Monaco, Matteo Romano Cantagalli, Matt Daggett, Alessandro Carrozzo, Alessandro Annibaldi, Natale Criseo, Luca Labianca, Andrea Ferretti

**Affiliations:** Sant’Andrea University Hospital, La Sapienza University of Rome, Rome, Italy

## Abstract

Anterior cruciate ligament (ACL) injuries among young patients have increased in recent years. The purpose of this study was to present a physeal-sparing intra- and extra-articular reconstruction using semitendinosus and gracilis tendons autograft. In recent years, the management of these injuries in the pediatric population has become increasingly surgical to restore knee function and reduce the risk of meniscal and chondral injury due to persistent knee instability. However, this is a population at high risk for ACL graft rupture, but it can be lowered by an addition of lateral extra-articular tenodesis (LET). This study shows the pearls and pitfalls of an arthroscopic physeal-sparing ACL reconstruction combined with a concomitant LET using hamstrings autograft.

In recent years, the incidence of anterior cruciate ligament (ACL) injuries among young patients has increased in conjunction with an increase in youth competitive sports.^1^^,^^2^ There has been a concomitant increase in 10.13039/100006307ACL reconstruction procedures in patients with open physis with a goal to avoid secondary damage to cartilage and meniscal structures.^3^, ^4^, ^5^ The surgical procedure is chosen depending on the growth stage estimated through skeletal age and Tanner’s staging of sexual maturation.^6^^,^^7^ There are 3 common techniques, including physeal sparing, partial transphyseal, and complete transphyseal.^8^ The physeal-sparing approach uses an autologous graft as the surgical treatment of choice for the prebubescent patients in Tanner I or II of development with a significant growth expected.^9^, ^10^, ^11^, ^12^ Recent literature also supports the need for adding a lateral extra-articular tenodesis (LET) concomitant to ACL reconstruction in skeletally immature patients to achieve best results in terms of rerupture rates.^13^, ^14^, ^15^

The Micheli and Kocher^9^ technique is the most common and published technique among the physeal-sparing procedures. The authors described an over-the-top, iliotibial band-based ACL reconstruction + lateral extra-articular tenodesis. The iliotibial band is harvested, and tibial attachment at Gerdy’s tubercle is preserved. The graft is tubularized and brought through the over-the-top position at the femoral side and the over-the-front position under the intermeniscal ligament and, thus, allows a concomitant intra- and extra-articular reconstruction.

The purpose of this study was to present a physeal-sparing intra- and extra-articular reconstruction using semitendinosus and gracilis tendons autograft.

## Surgical Technique

This technical note presents an arthroscopic physeal-sparing ACL reconstruction with a concomitant LET using a hamstring autograft. Pearls and pitfalls of this technique are presented in Table 1.

**Table 1:** Pearls and of Arthroscopic Physeal Sparing ACL Reconstruction with Semitendinosus and Gracilis Tendons

	Pearls
Preoperative	
	It is essential that the child undergoing surgery and its relatives are psychologically prepared to sustain the surgery and the postoperative rehabilitation.
Intraoperative	
	It is important to accurately harvest the semitendinosus and gracilis as close to the myotendinous junction, so that their full length can be used.
	Need to ensure the graft passage under the intermeniscal ligament
	It is important to assess the need for notchplasty of the lateral femoral condyle.
	Control the positioning using C-arms of the anchors in order not to involve the physes
	Use a suture wire between needles to avoid aberrations of the LET.
Postoperative	
	Respect rehabilitation time and do not precipitate a return to sport until the recovery of muscle trophism and neuromuscular control.
	
	Pitfalls
Intraoperative	
	An insufficient harvesting of the semitendinosus and gracilis can make difficult femoral and tibial fixation.
	Hamstrings harvesting must be conducted properly and gently: one must avoid damaging the tibial insertion of the hamstrings, since it is the only tibial fixation of the ACL reconstruction
	Notchplasty of the lateral femoral condyle should not be overdone so as not damage perichondral ring.
	Take care to avoid impacting the spear into the bone, which could compromise the cortex and lead to poor fixation.

### Step 1: Positioning and Diagnostic Arthroscopy

**Fig 1:**
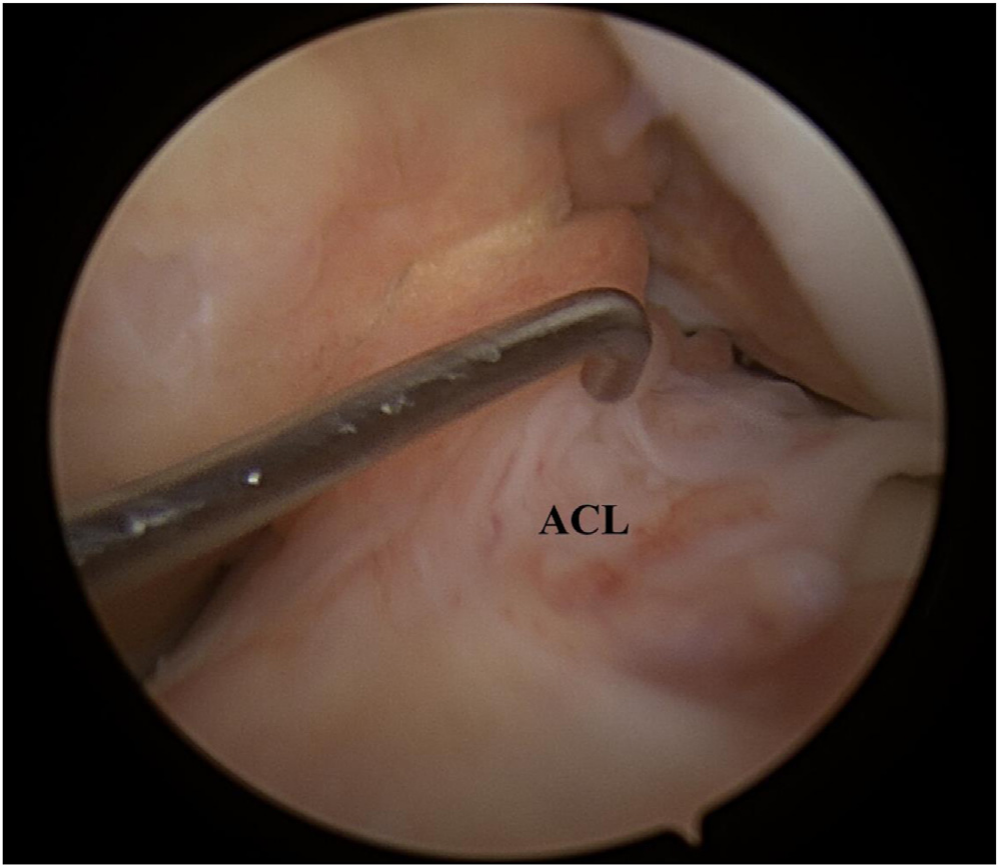
Arthroscopic view of the left knee. The arthroscopic probe is at the foot of the remnant of anterior cruciate ligament following the rupture. ACL, anterior cruciate ligament.

The procedure is performed with the patient under general anesthesia. The patient is placed supine on the operating table in the standard arthroscopy with a lateral support at the level of a padded tourniquet and foot roll positioned to maintain 90° of knee flexion. After preparation and draping of the leg, the arthroscopic portals are made. An anterolateral portal is established for the camera, and an anteromedial portal is established for instruments. The diagnostic arthroscopy is performed, and the status of the cartilage, the menisci, and the ligamentous structures are noted. The ACL lesion is identified, based on the arthroscopic appearance, and its instability is tested with an arthroscopic hook (Fig 1).

### Step 2: Shuttle Placement for Graft Passage for Combined Intra- and Extra-articular Reconstruction and Tendons Preparation

**Fig 2:**
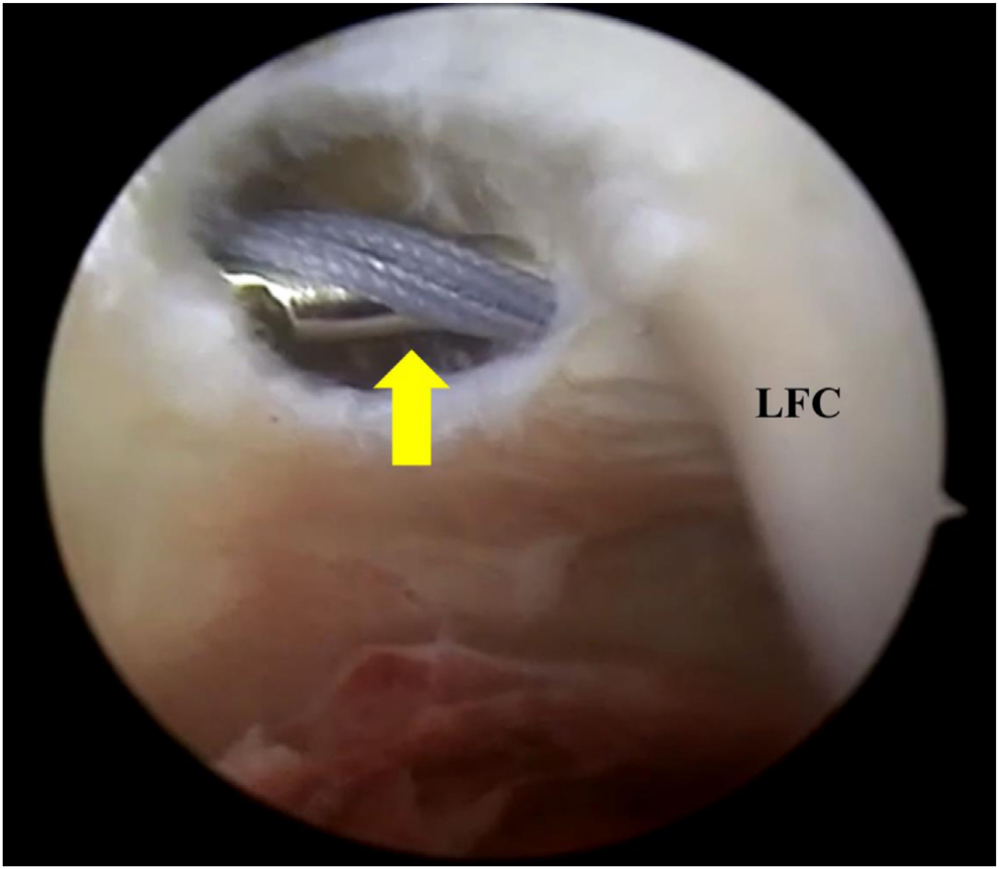
Arthroscopic view of the left knee. The scope is placed trans-notch to obtain a posterolateral view. The Kirschner wire with the no. 2 FiberWire (Arthrex) reaching the joint in the over-the-top position on the lateral femoral condyle. LFC, lateral femoral condyle. Yellow arrow denotes the Kirschner wire with no. 2 FiberWire (Arthrex).

Once the lesion has been identified and the ACL remnant is excised, a 6-cm hockey stick longitudinal incision is made proximal to the lateral femoral epicondyle extending to the Gerdy’s. The iliotibial band is incised longitudinally along its fibers and under arthroscopic control, a no. 2 FiberWire (Arthrex, Naples, FL) loop is inserted with a Kirschner wire until reaching the posterior aspect of the joint capsule in the over-the-top position below the lateral femoral condyle (Fig 2). A grasper is then inserted into the anteromedial portal, the no. 2 FiberWire (Arthrex) loop is retrieved.

A vertical 4-cm-long incision is made on the pes anserinus. The sartorius fascia is incised along its fibers, thus exposing the underlying semitendinosus and gracilis tendons, which are dissected and disconnected at the muscolotendinous junction with an open tendon stripper leaving their distal tibial insertion preserved. The 2 free ends of the tendons are debrided from the muscle tissue, and a Bunnel-style suture is placed in the free ends of each tendon with a no. 2 FiberWire (Arthrex).

### Step 3: Intra-articular Transposition of the Neoligament and Extra-articular Fixation

**Fig 3:**
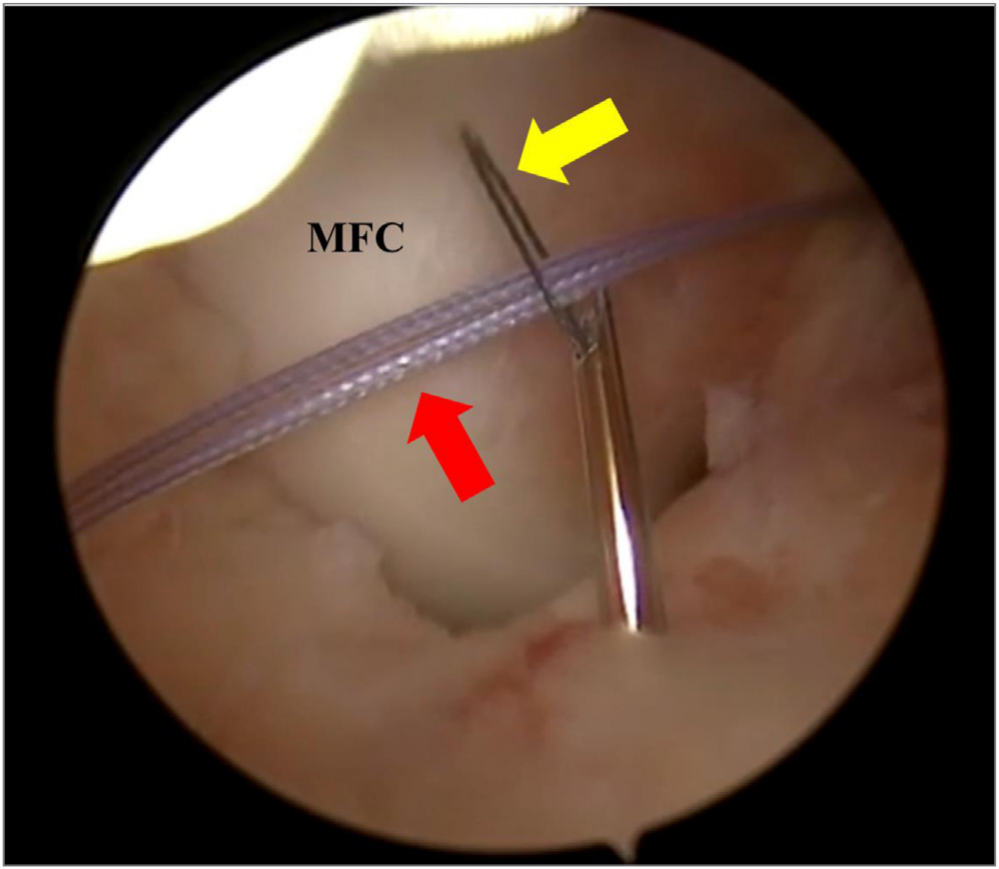
Arthroscopic view of a left knee. The scope is placed trans-notch to obtain a posterolateral view. A no. 2 FiberWire (red arrow; Arthrex) is passed into the Nitilon wire loop inside the Micro SutureLasso (yellow arrow; Arthrex). MFC, medial femoral condyle.

Through the tibial incision, a curved debridement rasp (Arthrex) is used to excavate a niche below the intermeniscal ligament. Subsequently, A curved Micro SutureLasso (Arthrex) is inserted into this groove on the anterior aspect of the proximal epiphysis of the tibia until a Nitilon wire loop is entered through the Micro SutureLasso (Arthrex) curved into the joint. The nitilon wire is retrieved by the anteromedial portal, the no. 2 FiberWire (Arthrex) acting as a suture passer, is passed through the Nitilon wire loop (Fig 3) and finally dragged externally with the Nitilon wire loop at the level of tibial skin incision.

**Fig 4:**
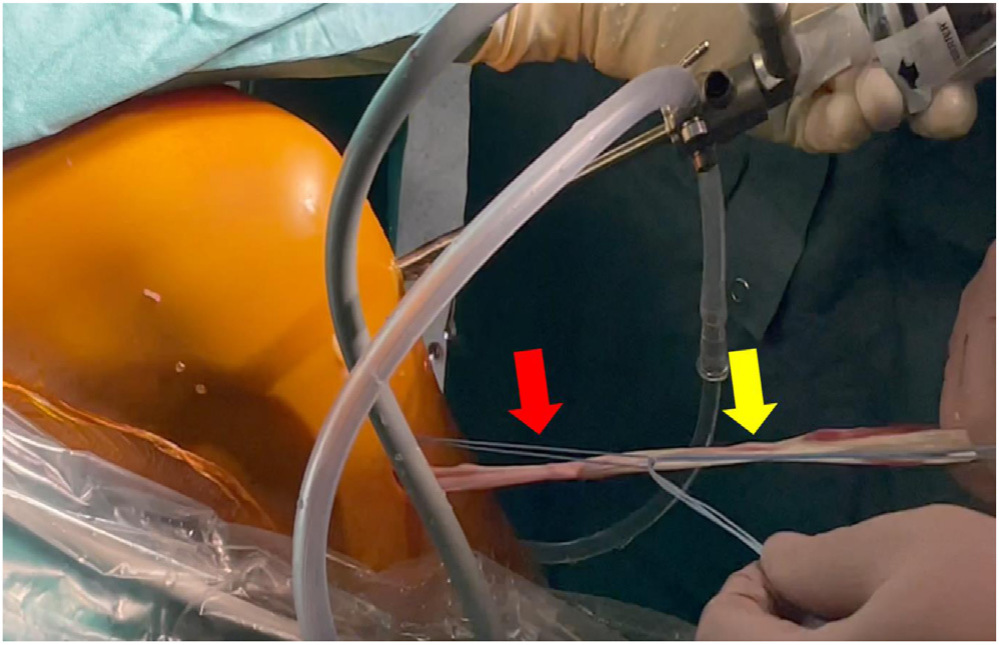
Medial view of a left knee. The semitendinosus and gracilis tendons distal attachments are preserved, and hamstrings continuous graft is used for both anterior cruciate ligament reconstruction and lateral extra-articular tenodesis. The graft is shuttled inside the knee from the niche under the intermeniscal ligament. Red arrow denotes no. 2 FiberWire (Arthrex), while yellow arrow denotes the graft.

**Fig 5:**
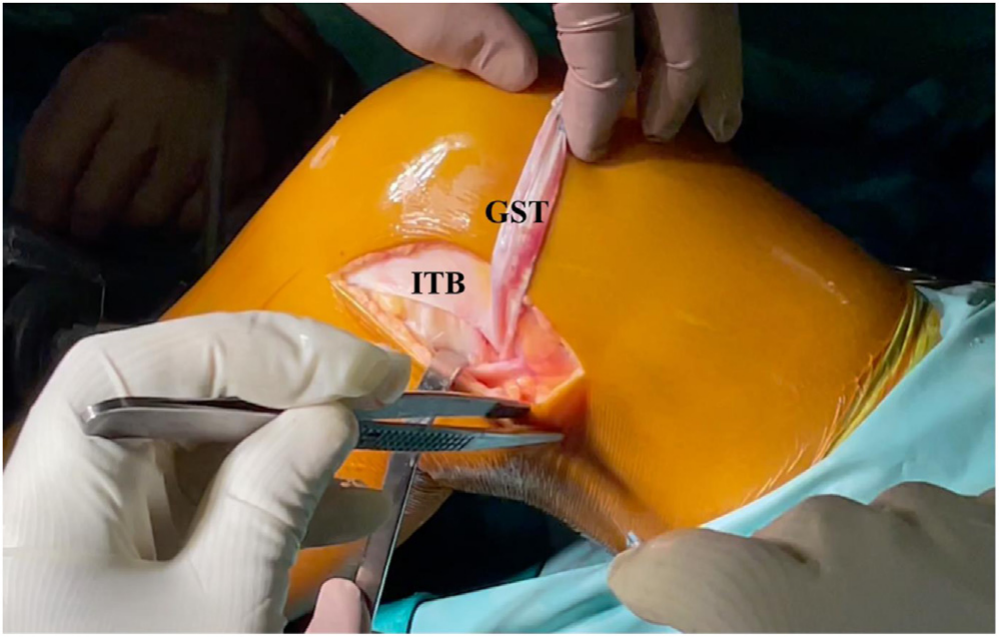
Lateral view of a left knee. The graft, composed of both semitendinosus and gracilis tendons, are shuttled outside the joint, passing between the lateral femoral condyle and the lateral gastrocnemius tendon. GST, gracilis and semitendinosus tendons free ends; ITB, iliotibial band.

The two tendons free ends are loaded inside the no. 2 FiberWire (Arthrex) shuttle (Fig 4). The no. 2 FiberWire (Arthrex, Naples, FL) shuttle is retrieved on the femoral side, and the tendons are passed under the intermeniscal ligament through the joint until they emerge from the lateral knee incision behind the lateral femoral condyle in the over-the-top position (Fig 5).

**Fig 6:**
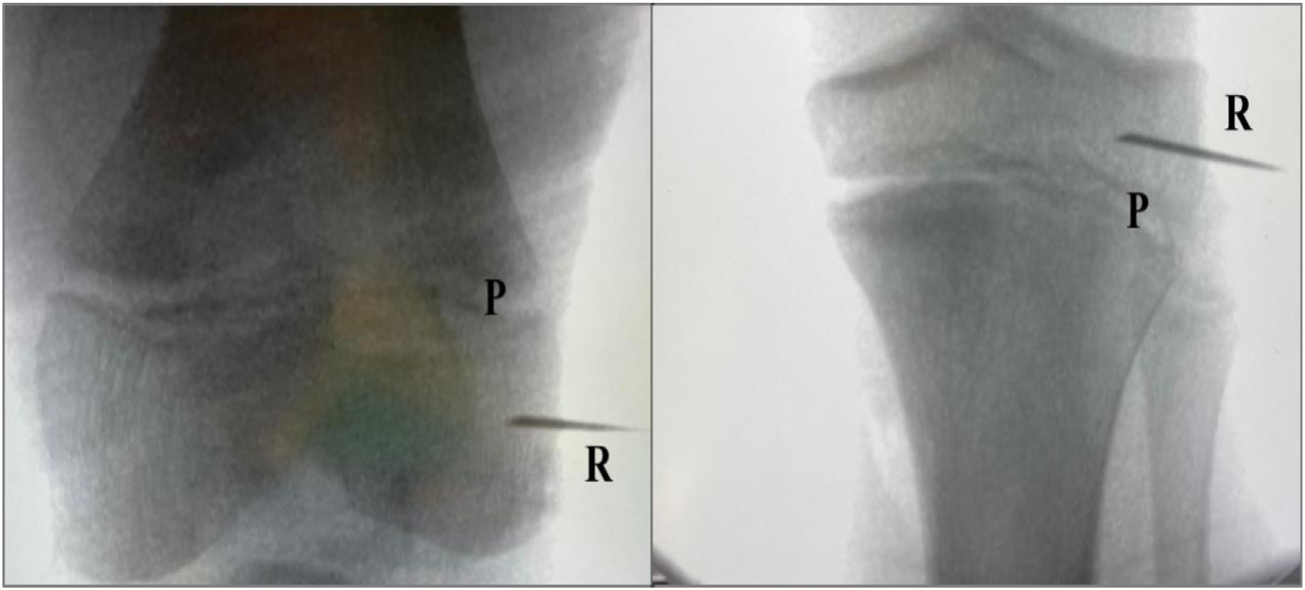
Using an image intensifier, the two soft anchors are placed in the epiphysis of the femur and tibia. Soft tissue anchor will provide fixation to the bone of the graft for both ACL reconstruction and LET. P, growth plate; R, needle repere.

Finally, the extra-articular fixation is made under fluroscopic control for taking care not to involve the physis. Two needles are placed as landmarks on the lateral femoral cortex and on the lateral aspect of the tibia at the level of the Gerdy’s tubercle in order to fix the intra-articular graft (femoral fixation) and to perform a lateral tenodesis (tibial fixation) (Fig 6). Then, the tension of the LET through the entire range of motion is tested with the use of a suture wire between the two needles.

Needles are removed, and a 1.8-mm bone socket using a FiberTak soft anchors drill (Arthrex) was made for both femur and tibia.

**Fig 7:**
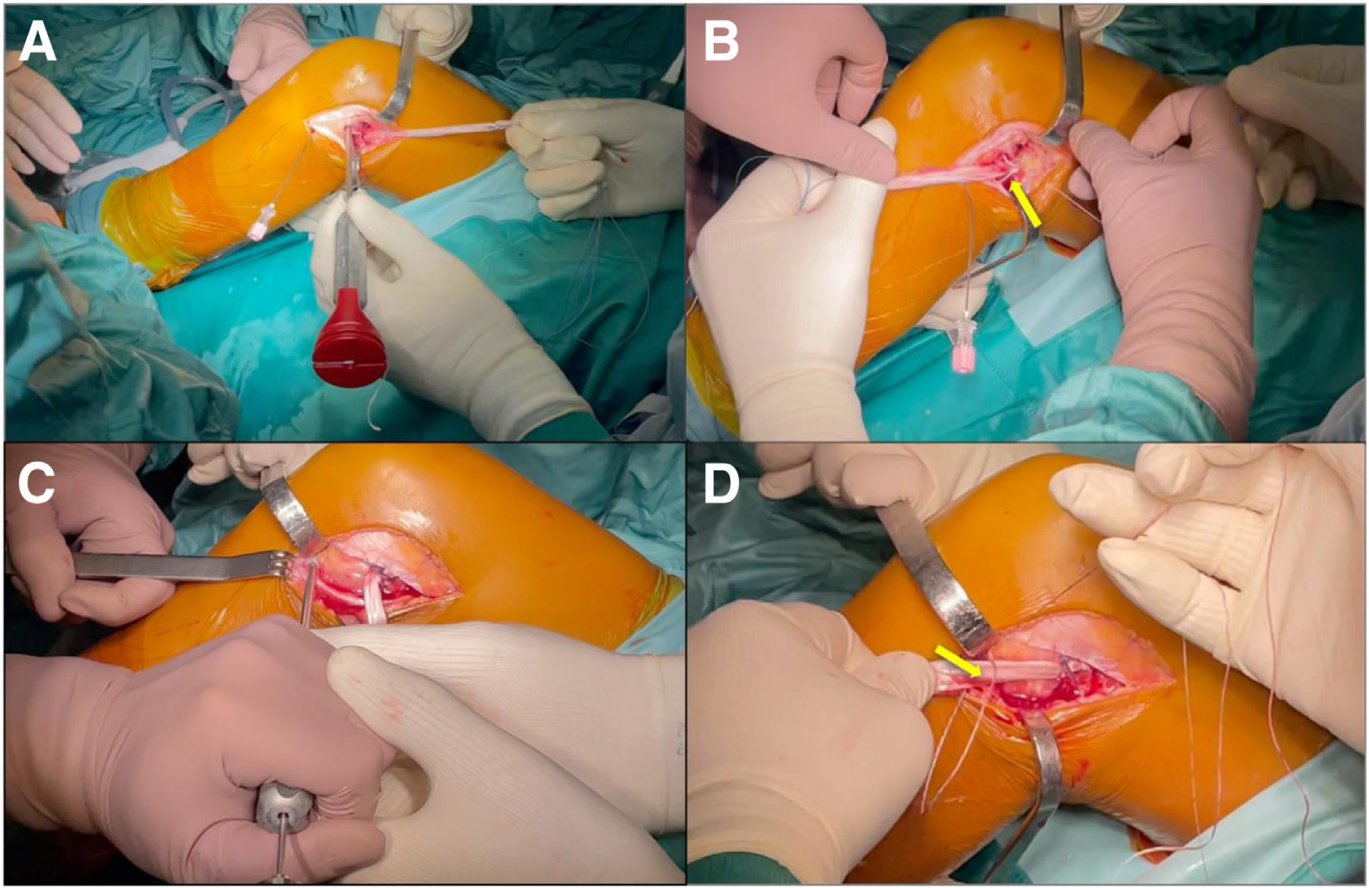
Lateral view of a left knee. The extra-articular fixation of the graft is performed with the two Knotless FiberTak soft anchors (Arthrex). (A) The placement of an anchor on the epiphysis of lateral femoral condyle. (B) The fixation of the graft on the lateral femoral cortex; yellow arrow shows the soft anchors fixing the graft. (C) Preparation of the 1.8-mm bone socket for the soft anchors on the lateral aspect of the epiphysis of the proximal tibia. (D) The fixation of the graft on the lateral aspect of the tibia; yellow arrow denotes the Knotless FiberTak soft anchors (Arthrex) fixing the graft.

**Fig 8:**
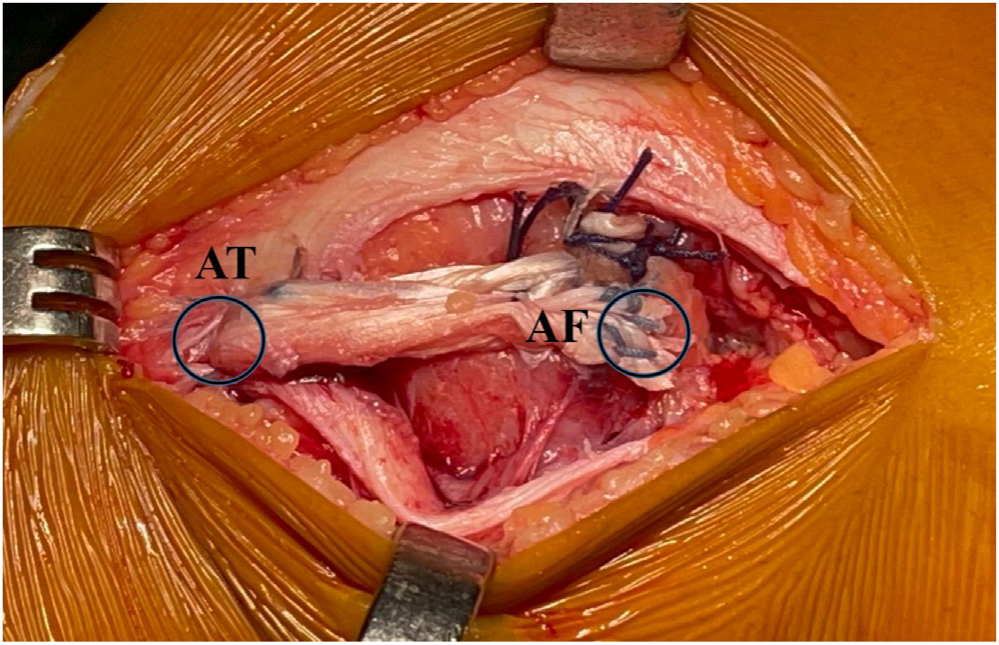
Lateral view of a left knee. Fixation of the hamstrings tendons graft at lateral distal femoral and lateral proximal tibia epiphyses with the two Knotless FiberTak soft anchors (Arthrex). AF, anchor on the lateral femoral cortex; AT, anchor on lateral aspect of the tibia.

The first anchor is inserted between the physis and the articular face of the lateral femoral condyle, and it is used to secure the graft to the lateral femoral cortex (Fig 7, A and B). Thereafter, with the second anchor, the graft is fixed on the lateral aspect of the tibia above the Gerdy’s tubercle (Fig 7, C and D). The final appearance of the lateral articular tenodesis is shown in Fig 8.

**Fig 9:**
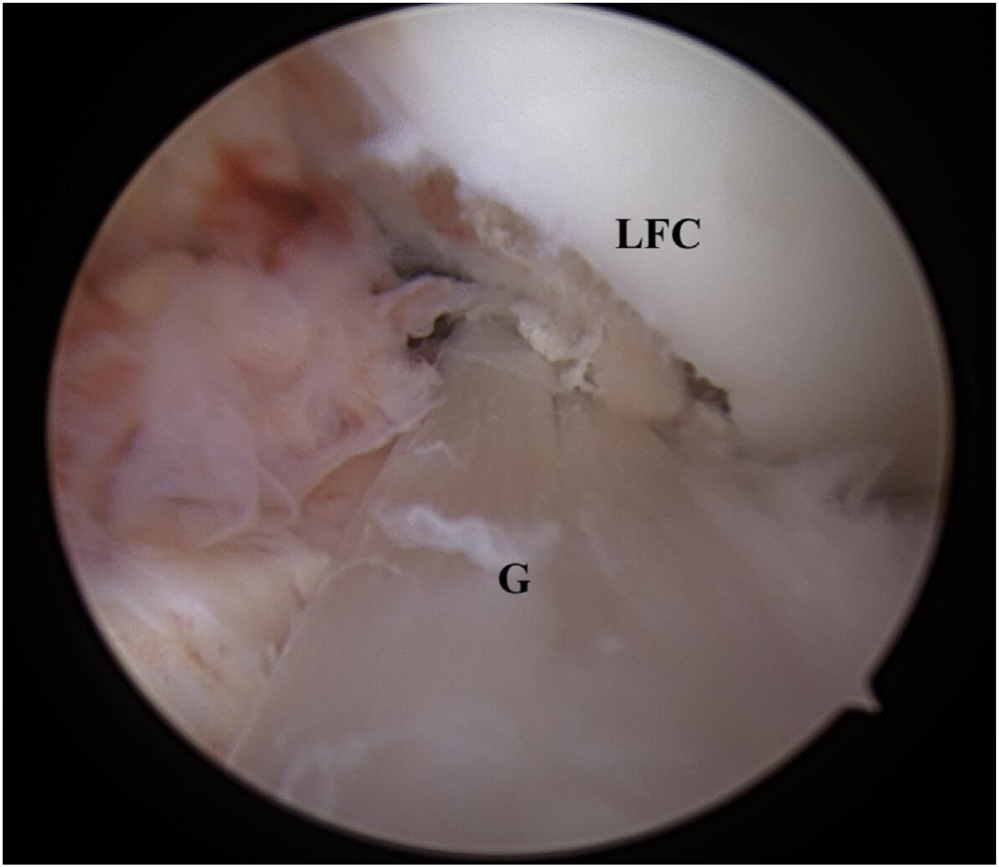
Intra-articular, arthroscopic view of a left knee after anterior cruciate ligament reconstruction using the continuous semitendinosus and gracilis tendons autograft. G, graft; LCF, lateral femoral condyle.

A minimal notchplasty of the lateral femoral condyle is performed to provide the graft with the right intra-articular position (Fig 9). Finally, the tourniquet is deflated, and hemostasis is achieved. The iliotibial tract defect is closed on the proximal part, and meticulous suturing of deep layers and the skin is performed. A standard sterile surgical dressing and a moderately compressive elastic bandage are applied to the knee, to include the foot and the leg. A brace is applied with the knee locked in full extension.

## Postoperative Rehabilitation

**Fig 10:**
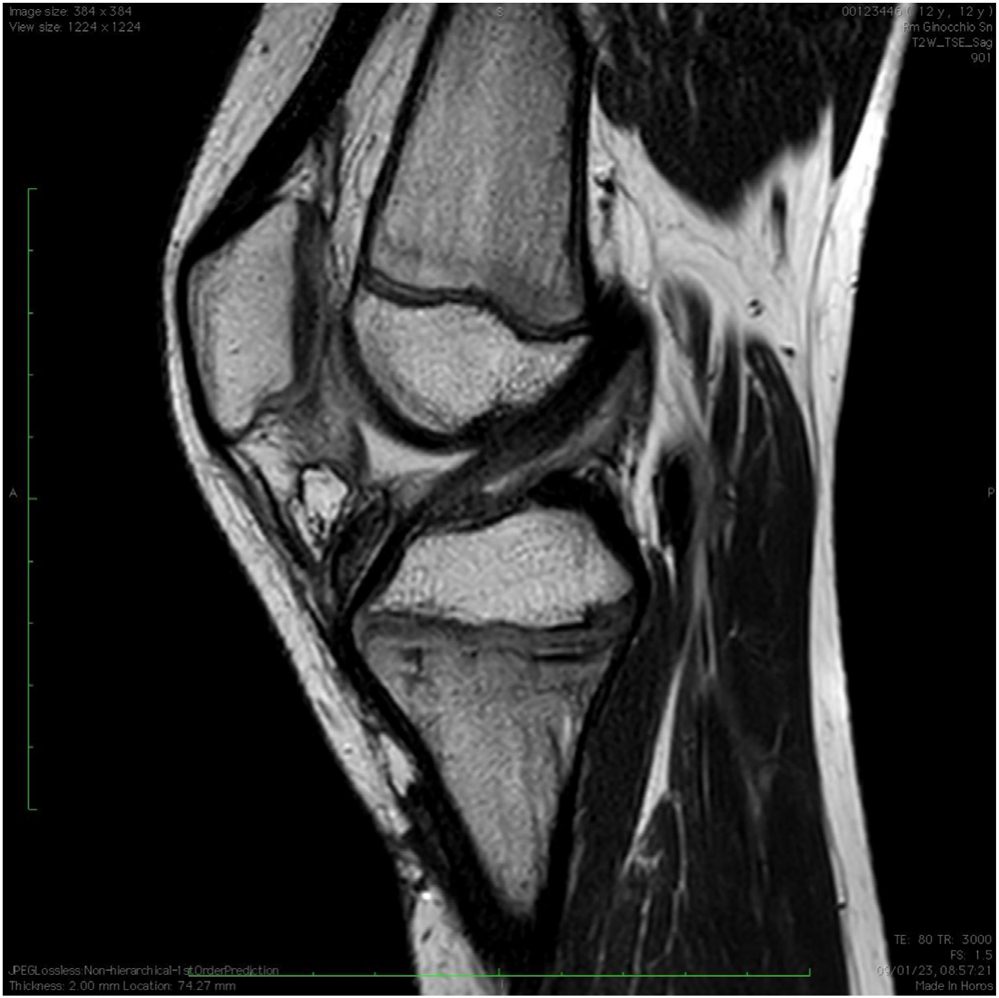
A sagittal, STIR (short-tau-inversion-recovery) sequence magnetic resonance imaging scan of a combined intra-articular anterior cruciate ligament (ACL) reconstruction and lateral extraarticular tenodesis with the described technique. This sequence shows the spearing of the femoral and tibial growth plates and good signal intensity and morphology of the ACL graft at 6 months after reconstruction.

A knee brace is applied for 6 weeks: full extension for 2 weeks, and subsequently, ROM is unlocked. Immediately after surgery, weight-bearing is allowed, as tolerated, with the aid of crutches, and isometric quadriceps exercises are started. At 2 weeks postoperatively, the patient begins active and passive flexion, which is limited to 90° until the 4th week, at which time, progressive range of motion exercises are encouraged. The brace is removed at the 6th week, and the weight-bearing without crutches is allowed. At 2 months after surgery, a quadriceps-strengthening program, as well as proprioceptive rehabilitation, are prescribed. Strengthening of the hamstrings is not allowed until 2 months postoperatively. Running is permitted after 4 months. An MRI is routinely prescribed at 6 months postsurgery (Fig 10) before starting sport-related training and return to play is allowed at 9 months postoperatively.

## Discussion

There are many advantages of the described technique. First, this is a physeal-sparing procedure and allows avoidance of the damage that tunnels would cause to the physis, which makes this technique particularly suitable for Tanner I-II patients. This is a nonanatomical ACL reconstruction as the graft is in the over-the-top position at the femur and under the intermeniscal ligament at the tibia. Although this may be a downside of the current technique, this configuration has already been used in several clinical trials using iliotibial band (ITB).^9^^,^^16^^,^^17^ Kocher et al.^16^ reported the outcomes of a large series of patients receiving ACLR+LET using this configuration. They reported the results of 240 patients who, at a mean follow-up of 26 months, returned to sport in 96.5% with a graft rupture rate of 4% and excellent results at PROMs: on average 93.4 at Lysholm and 93.3 at Pedi-IKDC. Willimon et al.^17^ conducted a study using the same technique as ACLR+LET on a smaller population of 22 patients at a mean 36-month follow-up, reporting a return to sport of 78.9% and 13.6% graft ruptures. Even in this population, however, the results of PROMs were more than satisfactory: an average of 95 at Lysholm and 96.5 at Pedi-IKDC. The technique presented in the current article is intended to reproduce the procedure described by Micheli and Kocher but using hamstring graft instead of a strip of ITB.

The use of the hamstrings graft instead ITB can have some advantages. The first is a less extensive skin incision limited to a small incision on the pes anserinus for tendons harvesting a 6-cm hockey stick incision on the femoral side. The second is the use of hamstrings tendons graft that have a wider literature supporting their use as intra-articular graft. Further, hamstrings tendons have better biomechanical properties in comparison with ITB.^18^

**Fig 11:**
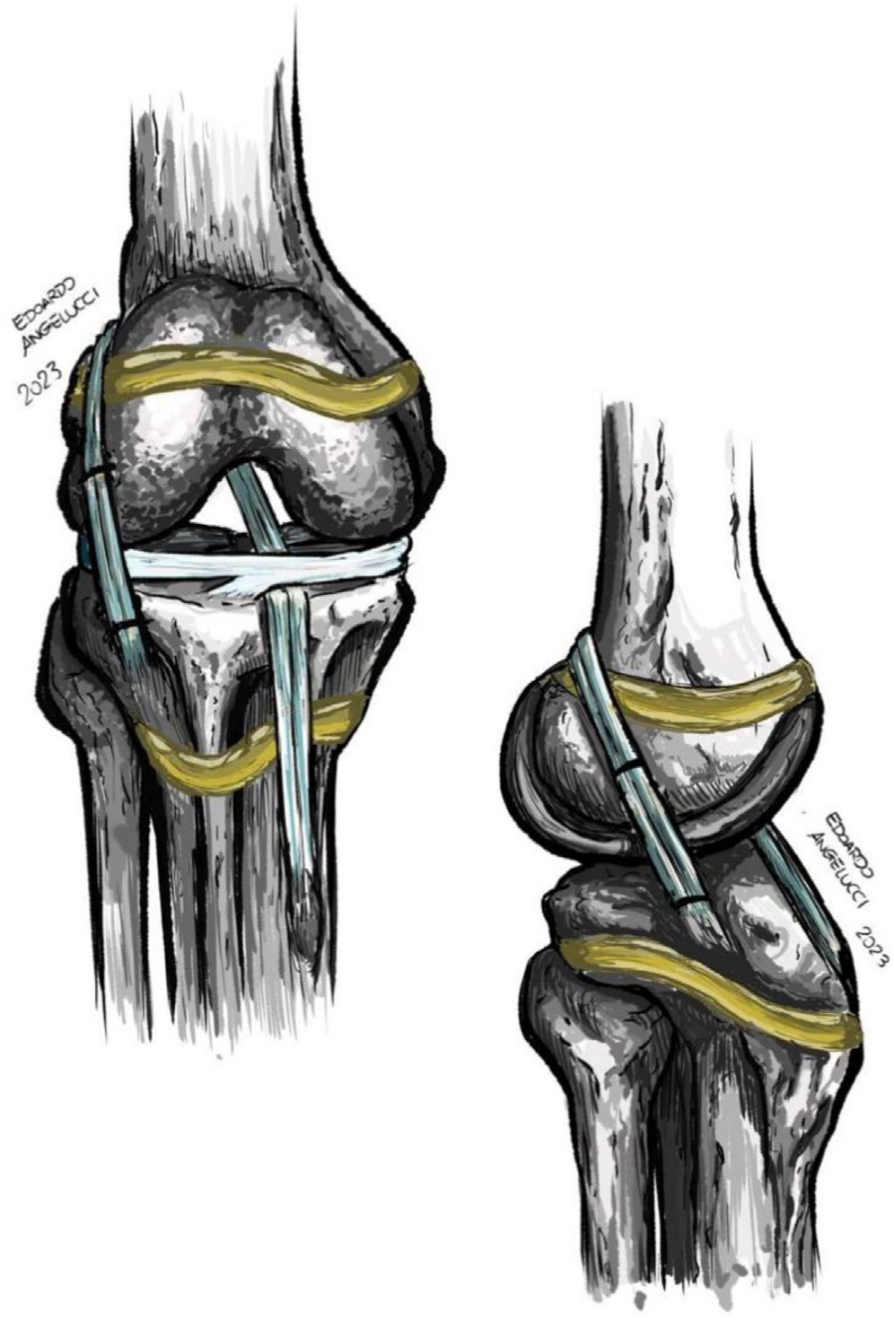
Illustration of the described technique, demonstrating the physeal sparing, combined anterior cruciate ligament reconstruction, and lateral extra-articular tenodesis using semitendinosus and gracilis tendons autograft.

This technique allows a LET to be performed with a fixation that does not cross the physes unlike other techniques, such as the as the Modified Lemaire. Foissey et al.^19^ conducted a retrospective, single-center study that investigated the outcomes of ACL reconstruction in growing children combined with 2 different lateral extra-articular procedures (anatomic reconstruction with a gracilis graft or modified Lemaire technique with a strip of fascia lata). With an average follow-up of 57 months, the study included 39 patients (40 ACLs), aged 13.8 ± 1.4 years. Results showed 1 graft failure (2.6%), 2 cases (5.4%) of femoral overgrowth, which required intervention in 1 patient, and significant improvements in side-to-side anterior laxity. However, this fixation could theoretically lead to an increase in tensile forces on the physis, resulting on a physeal compression. Although a direct correlation between the execution of a LET and growth disturbances has not been proven, the current technique eliminates the potential risk of deformity of the operated lower limb due to excessive compression exerted at the level of the physis. (Fig 11)

Another advantage is the use of a nonmetallic fixation. The use of soft-tissue anchors reduces the risk of the hardware being symptomatic, thus reducing the risk of reintervention for hardware removal. The use of a spear allows one to be precise in the trajectory of the bone socket and its concurrent use of C-arms allows for control of the direction and depth of the drill. Also, the spear allows the anchor to be placed in the desired direction, maintaining the drill trajectory. Once the suture is passed and shuttled into the locking mechanism, tension of the graft fixation can be controlled and adjusted under direct visualization, without risk of knot loosening.

In conclusion, this technique represents a physeal-sparing ACL reconstruction combined with a lateral extra-articular tenodesis using hamstrings tendons autograft. The continuous intra-articular and the extra-articular graft is fixed with all suture anchors respecting the femoral and tibial physis integrity.

## Disclosure

The authors report the following potential conflicts of interest or sources of funding: A.F., E.M., and M.D. report grants, consulting fees, equipment support, and speaker fees from Arthrex. Full ICMJE author disclosure forms are available for this article online, as supplementary material.

## Supplementary Data

ICMJE author disclosure forms

Video 1 This video presents a reproducible one-stage technique for combining arthroscopic physeal sparing anterior cruciate ligament. Reconstruction and lateral extraarticular tenodesis with semitendinosus and gracilis tendons autografts is shown.
